# Autoimmune Pancreatitis Type 1 with Biliary, Nasal, Testicular, and Pulmonary Involvement: A Case Report and a Systematic Review

**DOI:** 10.3390/jcm12196340

**Published:** 2023-10-03

**Authors:** Mourad Kourie, Darko Bogdanovic, Kamran Mahmutyazicioglu, Sam Ghazi, Nikola Panic, Eva Fjellgren, Laila Hellkvist, Tomas Thiel, Anders Kjellman, Nikolaos Kartalis, Olof Danielsson, Lara Dani, J.-Matthias Löhr, Miroslav Vujasinovic

**Affiliations:** 1 Department of Medicine, Vrinnevisjukhuset, 603 79 Norrköping, Sweden; mourad.kourie@hotmail.com; 2 Department of Urology, Karolinska University Hospital, 141 86 Stockholm, Sweden; darko.bogdanovic@regionstockholm.se (D.B.); tomas.thiel@regionstockholm.se (T.T.); anders.kjellman@regionstockholm.se (A.K.); 3 Department of Radiology, Karolinska University Hospital, 141 86 Stockholm, Sweden; kamran.mahmutyazicioglu@regionstockholm.se (K.M.); nikolaos.kartalis@regionstockholm.se (N.K.); 4 Department of Pathology, Karolinska University Hospital, 141 86 Stockholm, Sweden; sam.ghazi@regionstockholm.se (S.G.); olof.danielsson@regionstockholm.se (O.D.); 5 Digestive Endoscopy Unit, University Clinic “Dr Dragisa Misovic-Dedinje”, 11000 Belgrade, Serbia; nikola.panicmail@gmail.com; 6 Medical Library, Karolinska University Hospital, 171 76 Stockholm, Sweden; eva.fjellgren@regionstockholm.se; 7 Department of Ear, Nose and Throat, Karolinska University Hospital, 171 76 Stockholm, Sweden; laila.hellkvist@regionstockholm.se; 8 Department of Clinical Science, Intervention, and Technology (CLINTEC), Karolinska Institute, 141 86 Stockholm, Sweden; matthias.lohr@ki.se; 9 Department of Rheumatology, Karolinska University Hospital, 141 86 Stockholm, Sweden; lara.dani@regionstockholm.se; 10 Department of Upper Abdominal Diseases, Karolinska University Hospital, 141 86 Stockholm, Sweden; 11 Department of Medicine, Huddinge, Karolinska Institute, 141 86 Stockholm, Sweden

**Keywords:** IgG4-related disease, immunoglobulin G4, autoimmune, pancreatitis, orchitis, nasal polyps

## Abstract

Introduction: Immunoglobulin G4-related disease (IgG4-RD) is an immune-mediated condition associated with fibroinflammatory lesions that can occur at almost any anatomical site. It often presents as a multiorgan disease that may mimic malignancy, infection, or other immune-mediated conditions. Autoimmune pancreatitis (AIP) type 1 is the most prominent manifestation of IgG4-RD in the digestive tract, with common extra-pancreatic inflammation. We present the first patient with AIP and involvement of the testicles and nasal cavity. Patient and methods: A case of a patient with AIP type 1 and other organ involvement (bile ducts, testicles, nasal polyps, and lungs) is described. Additionally, a systematic review of AIP type 1 with testicular and nasal involvement was conducted. Results: The systematic review found two cases of AIP type 1 with testicular involvement and 143 cases with AIP type 1 with nasal cavity involvement. None of them had both testicular and nasal involvement. Conclusions: This is the first case of AIP type 1 with other organ involvement, including testicular and nasal involvement, to be described. The number of patients with nasal and testicular involvement described in the literature is low. Creating awareness of this rare clinical condition is necessary, especially due to the very effective available treatment with corticosteroids and rituximab.

## 1. Introduction

**Key points:**

- Immunoglobulin G4 (IgG4)-related disease (IgG4-RD) is an immune-mediated condition associated with fibroinflammatory lesions that can occur at almost any anatomical site. Autoimmune pancreatitis (AIP) type 1 is the most prominent manifestation of IgG4-RD in the digestive tract with common extra-pancreatic inflammation.
- It often presents as a multiorgan disease that may mimic malignancy, infection, or other immune-mediated conditions.
- A case of a patient with AIP type 1 and other organ involvement (bile ducts, testicles, nasal polyps, and lungs) is described.
- Additionally, a systematic review of AIP type 1 with testicular and nasal involvement was conducted.

Immunoglobulin G4 (IgG4)-related disease (IgG4-RD) is an immune-mediated condition associated with fibroinflammatory lesions that can occur at almost any anatomical site. It often presents as a multiorgan disease that may mimic malignancy, infection, or other immune-mediated conditions [1]. Since it was first recognized as a distinct disease in 2003, it has become clear that the disease can affect virtually any organ, with strong predilections for certain organs, such as the major salivary glands (e.g., submandibular, parotid, sublingual), the orbits and lacrimal glands, the pancreas and biliary tree, the lungs, the kidneys, the aorta and retroperitoneum, the meninges, and the thyroid gland (Riedel’s thyroiditis) [1,2,3]. Autoimmune pancreatitis (AIP) was first described in 1961 in a case report of a patient with immune-related cholangitis and Mikulicz disease [4], but the name AIP was coined in 1995 [5]. It soon became obvious that the majority of patients with AIP do not have isolated disease of the pancreas; rather, “other organ involvement” is present in the most prevalent form of AIP, classified as type 1, i.e., IgG4-related (AIP type 2 is associated with inflammatory bowel disease) [6,7]. AIP type 1 is the most prominent manifestation of IgG4-RD in the digestive tract, with four key histological features (lymphoplasmacytic infiltration, storiform fibrosis, obliterative phlebitis, and increased numbers of IgG4+ plasma cells), classical imaging features (parenchymal enlargement-“sausage-like” shape, peripancreatic edematous rim, and main pancreatic duct narrowing without upstream dilatation), and excellent response to corticosteroid treatment; however, relapsing is common, especially in patients with other organ involvement. The association of AIP type 1 with extra-pancreatic inflammation (e.g., kidneys, lungs, cardiovascular), as well as its response to different treatment regimens (including corticosteroids and rituximab), has already been published by our group [8,9,10]. This case report (the first in our cohort with the involvement of both testicles and nasal cavity) and systematic review aims to raise awareness, characterize the clinical and serologic features, and assess the outcomes for patients.

## 2. Methods

A case of a patient with autoimmune pancreatitis type 1 (IgG4 disease in the pancreas) and other organ involvement (bile ducts, testicles, nasal polyps, and lungs) is described. Additionally, a systematic review of AIP type 1 with testicular involvement and AIP type 1 with nasal involvement was conducted. 

## 3. Case Report

**Figure 1:**
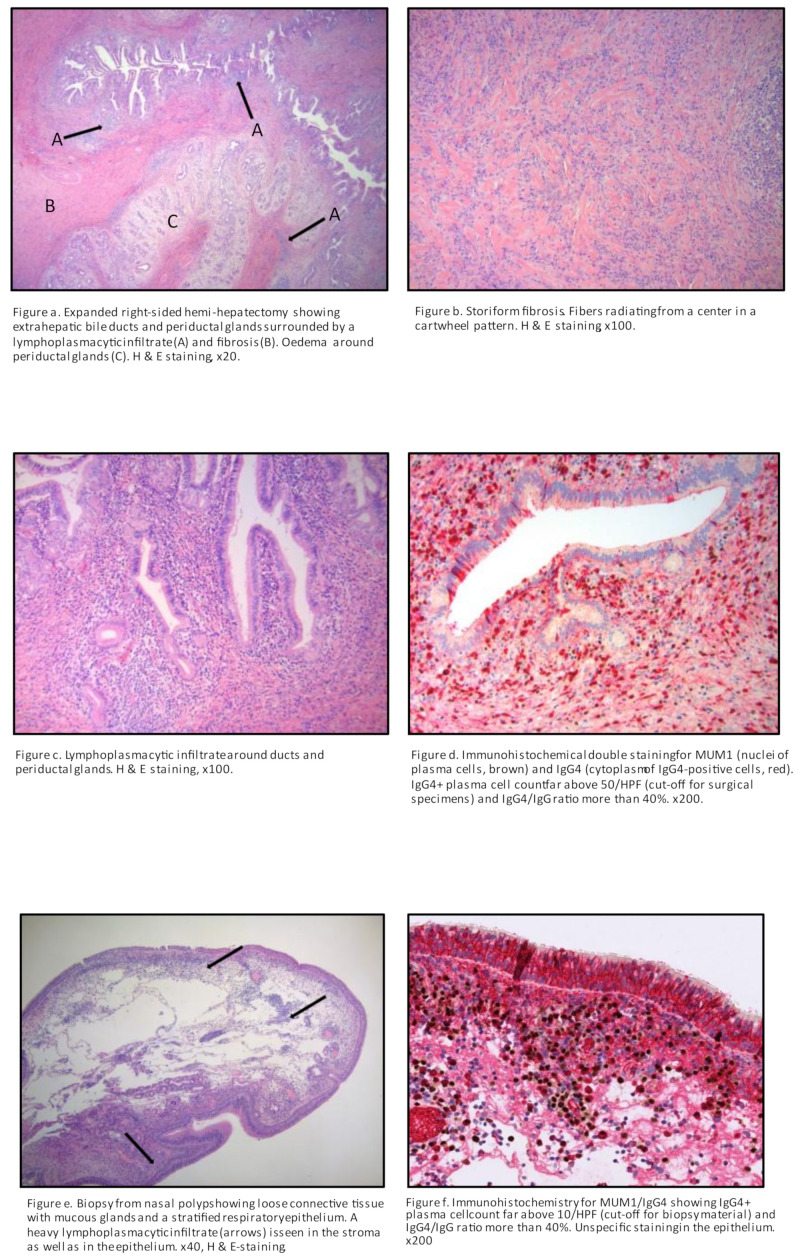
Histopathology after hemi-hepatectomy and biopsy of nasal polyps.

A 65-year-old male (former smoker, 10-year history of well-controlled arterial hypertension), presented at the urgent care unit of city hospital in July 2017 with a history of 15 kg of weight loss over a two-month period and painless jaundice that appeared two days before presentation (symptoms started while the patient was in another country, and he traveled home thereafter). The patient denied abdominal pain. Laboratory tests showed elevated liver function (bilirubin 281 μmol/L, AST 0.9 μkat/L, ALT 1.56 μkat/L, GGT 3.5 μkat/L, ALP 8.4 μkat/L). Computed tomography (CT) showed dilated intrahepatic bile ducts and a 3 cm suspected tumor in the liver hilus. An endoscopic retrograde cholangiopancreatography (ERCP) was performed on the same day and, in the endoscopist’s opinion, the lesion was suspected to be a perihilar tumor (Bismuth 3A). Two stents were placed (8.5 Fr, 12 cm), and a brushing cytology was performed (benign result). He was sent home and, after one week, was referred to a multidisciplinary team conference at our center: magnetic resonance imaging (MRI) and a new ERCP for cytology were recommended. An ERCP with stent replacement (12 Fr, 12 cm) and control cytology was performed in a city hospital one week after the first, and the cytology was benign again. After four days, the patient presented again at the urgent care unit at the city hospital with jaundice and bilirubin levels of 323 μmol/L. A new MRI was performed, which confirmed the previously suspected 3 cm tumor in the liver hilus. The reason for the new onset of jaundice was the non-optimal position of the bile-duct stent, which had slipped distally from stricture. The patient’s case was discussed again at a multidisciplinary team conference, and the decision was made to perform urgent liver surgery with curative intention. Surgery was performed at our center and included extended right-side hemi-hepatectomy, resection of lobus caudatus and extrahepatic bile ducts, lymphadenectomy in the liver hilum, and left side cholangiojejunostomy. A pathohistological analysis showed no signs of malignancy, but lymphoplasmacytic infiltration, storiform fibrosis, and strongly elevated IgG4 plasma cells (>50 per high power field), as well as a diagnosis of IgG4-related cholangitis, were confirmed (Figure 1). The serum IgG subclasses were analyzed after surgery, and showed elevated levels of total IgG (23.5 g/L; reference values: 6.7–14.5 g/L), IgG1 (12.5 g/L; reference values: 2.8–8.0 g/L), and IgG2 (10.4 g/L; reference values 1.15–5.7 g/L), but normal levels of IgG3 (0.33 g/L; reference values: 0.24–1.25 g/L) and IgG4 (0.24 g/L; reference values: 0.05–1.25). However, a control analysis after a few weeks showed strongly elevated IgG4 (10.8 g/L). The patient was referred to the rheumatology outpatient clinic for follow-up of IgG4-RD. No specific therapy was suggested because a control MRI in 2018 showed no signs of inflammation in abdominal organs, the laboratory tests (liver function test and inflammatory parameters) normalized, and the patient recovered well. A control MRI in 2020 showed signs of cholangitis and pancreatitis. The patient was referred to the pancreatology outpatient clinic and a routine work-up was performed, showing normal exocrine (fecal elastase-1 levels > 500 μg/g) and endocrine (normal glycated hemoglobin = HbA1c) pancreatic function and a normal serum nutritional panel (fat-soluble vitamins and trace elements). A dual-energy X-ray absorptiometry (DXA) showed osteopenia. 

**Figure 2:**
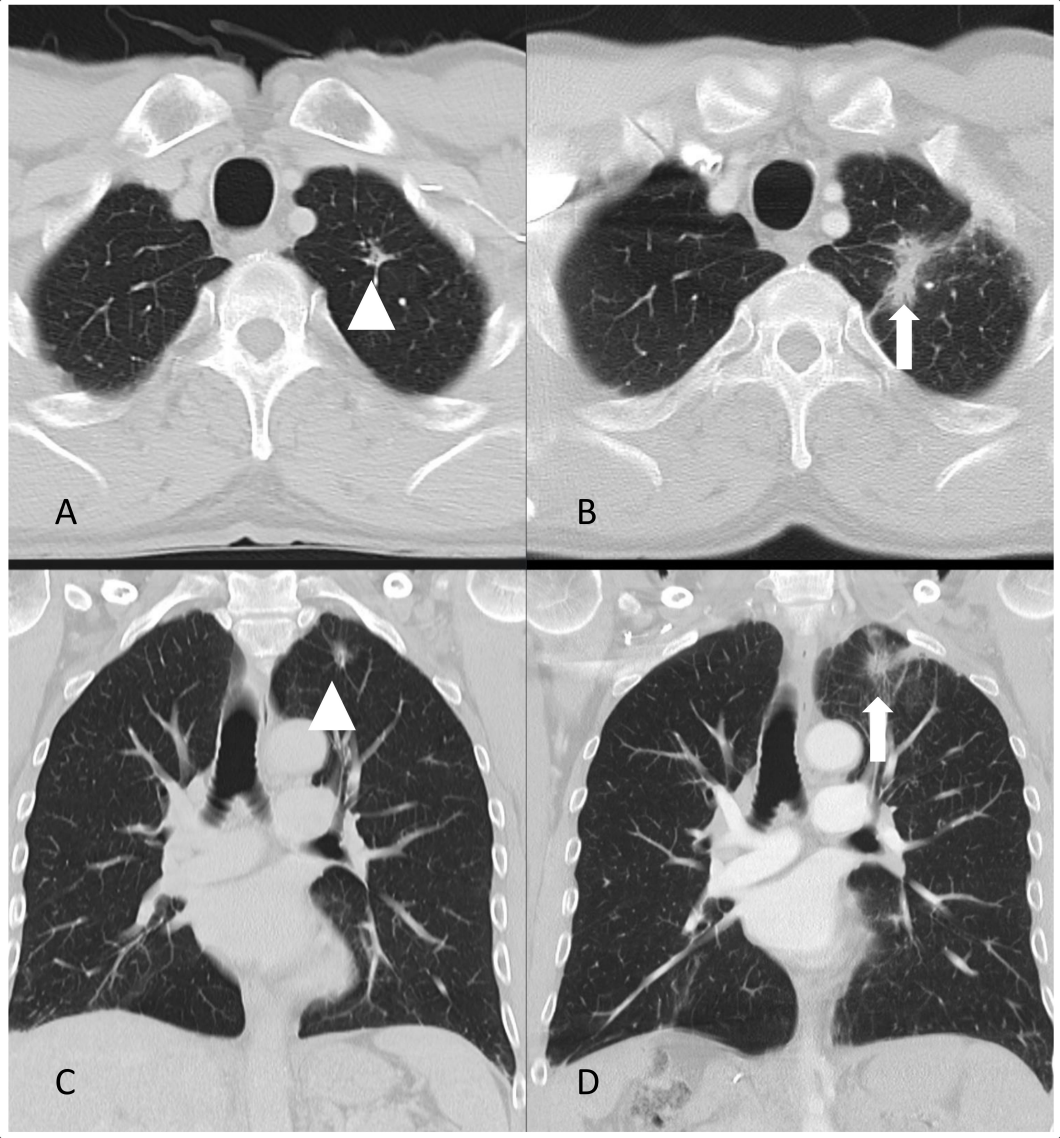
Axial (**A**,**B**) and coronal (**C**,**D**) CT images of the lungs in 2017 (**A**,**C**) and 2020 (**B**,**D**). The arrow in (**B**,**D**) indicates a spiculated infiltrate that had increased considerably in size since 2017, as indicated by the arrowhead in (**A**,**B**).

**Figure 3:**
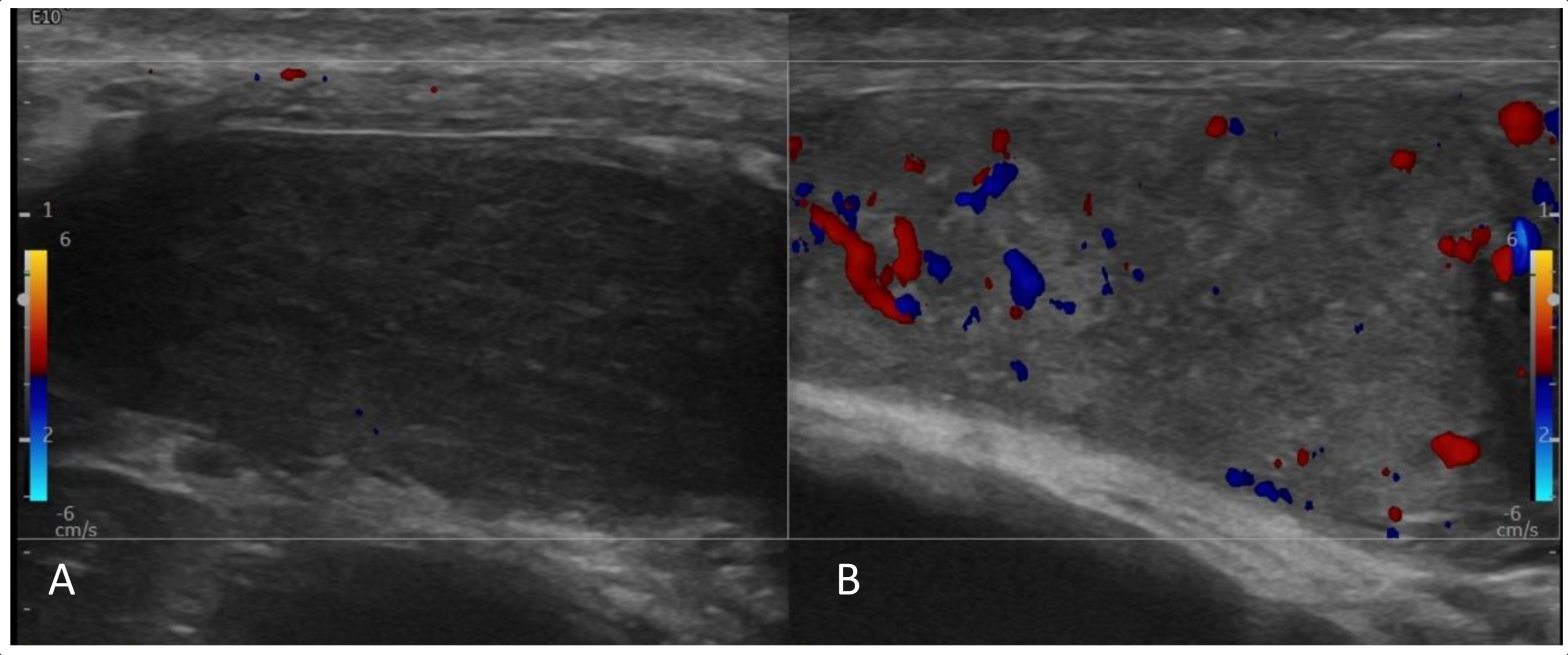
Ultrasound of testicles. Both testicles were atrophic (**A**,**B**). (**A**) The right testis was diffusely hypoechoic without a focal lesion. No detectable Doppler circulation in the right testicle. (**B**) Left testis had heterogeneous echogenicity. Doppler image shows an ill−defined hypoechoic area with decreased circulation in the center of the left testis.

**Figure 4:**
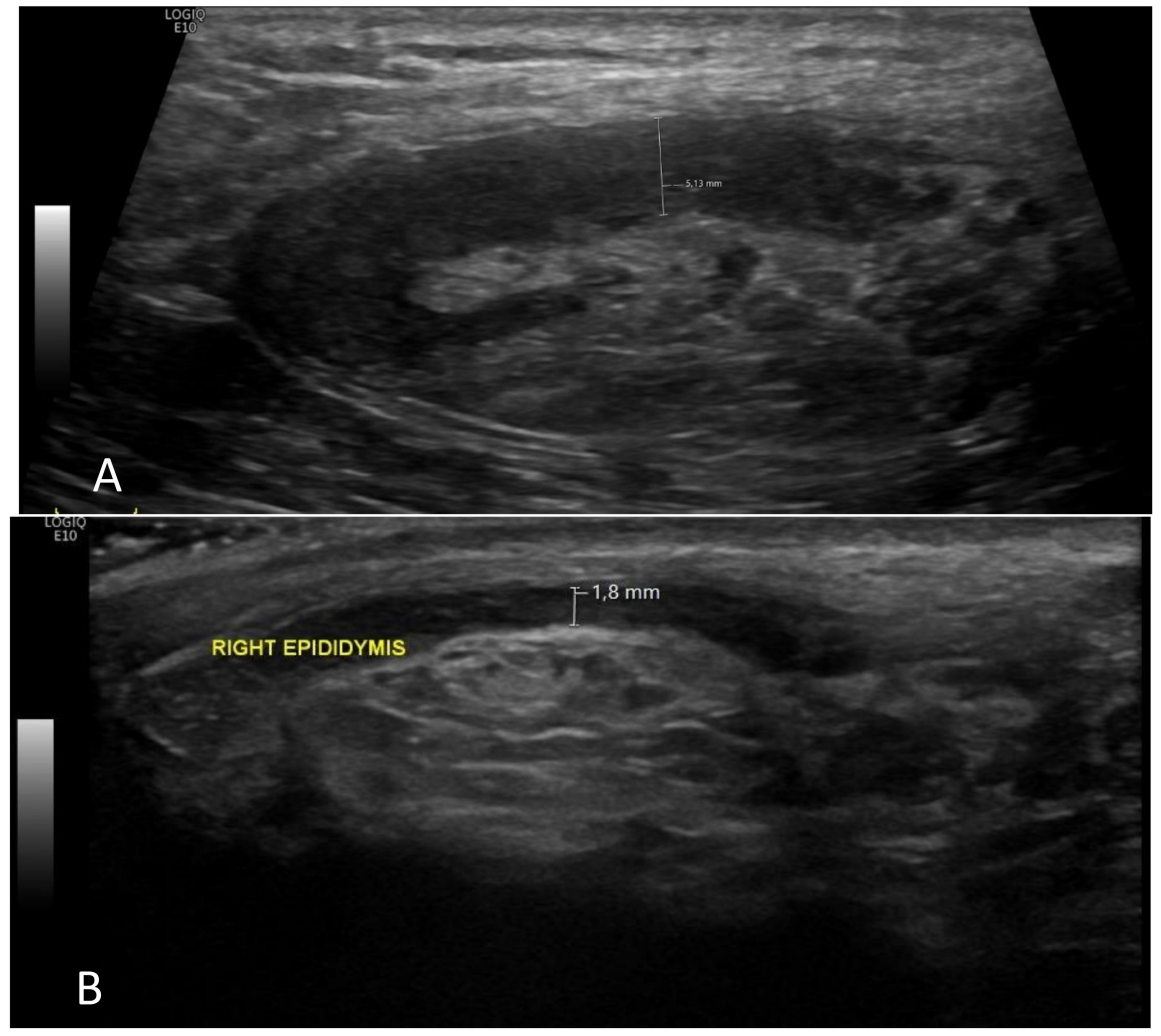
The right epididymis had normal volume on control ultrasound examination (**A**), but it was enlarged and heterogeneous before the treatment (6 months ago) (**B**).

**Figure 5:**
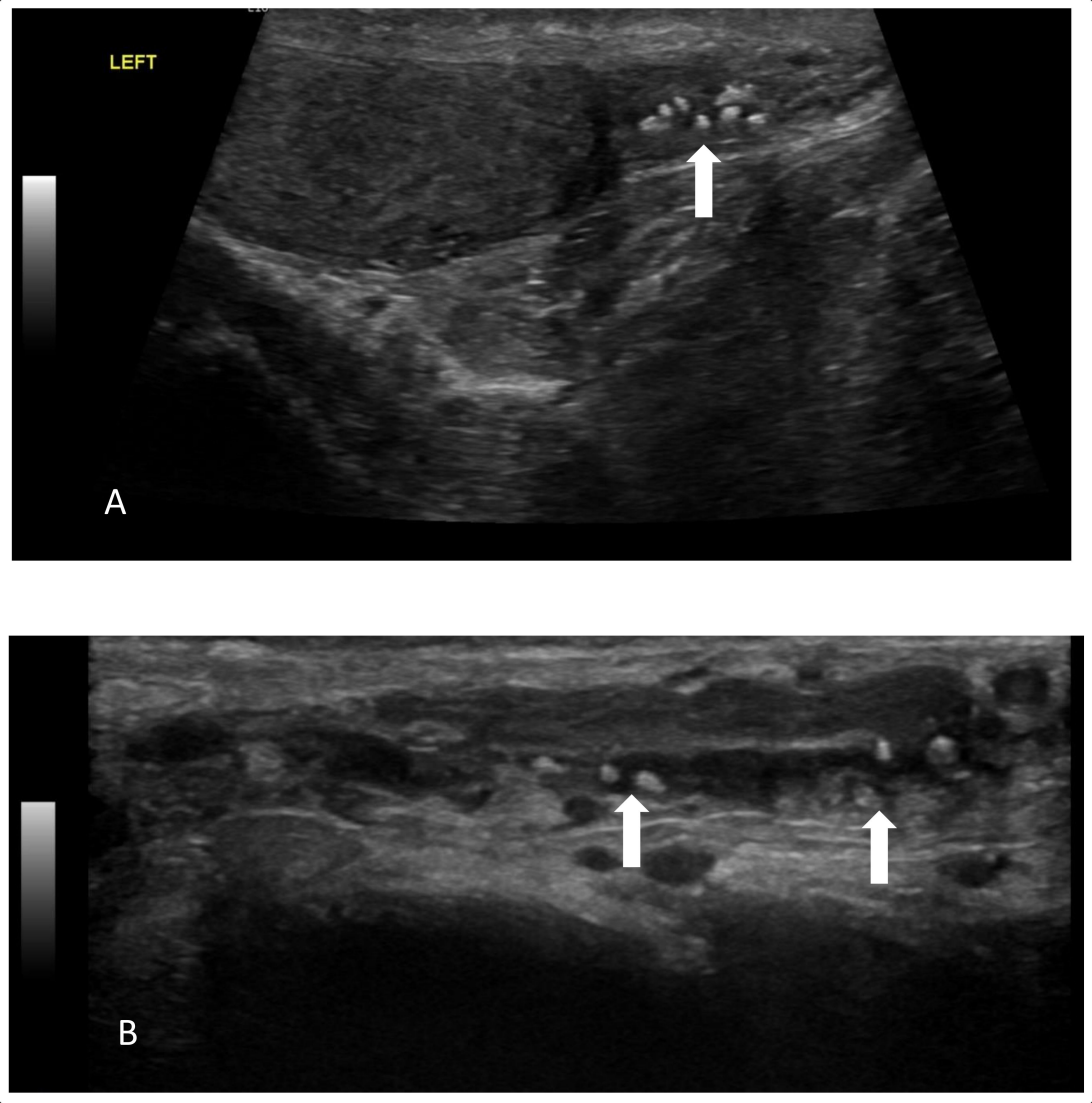
Multiple intraluminal calcifications (arrows) in the caudal (**A**) and scrotal segments of left vas deferens (**B**).

**Figure 6:**
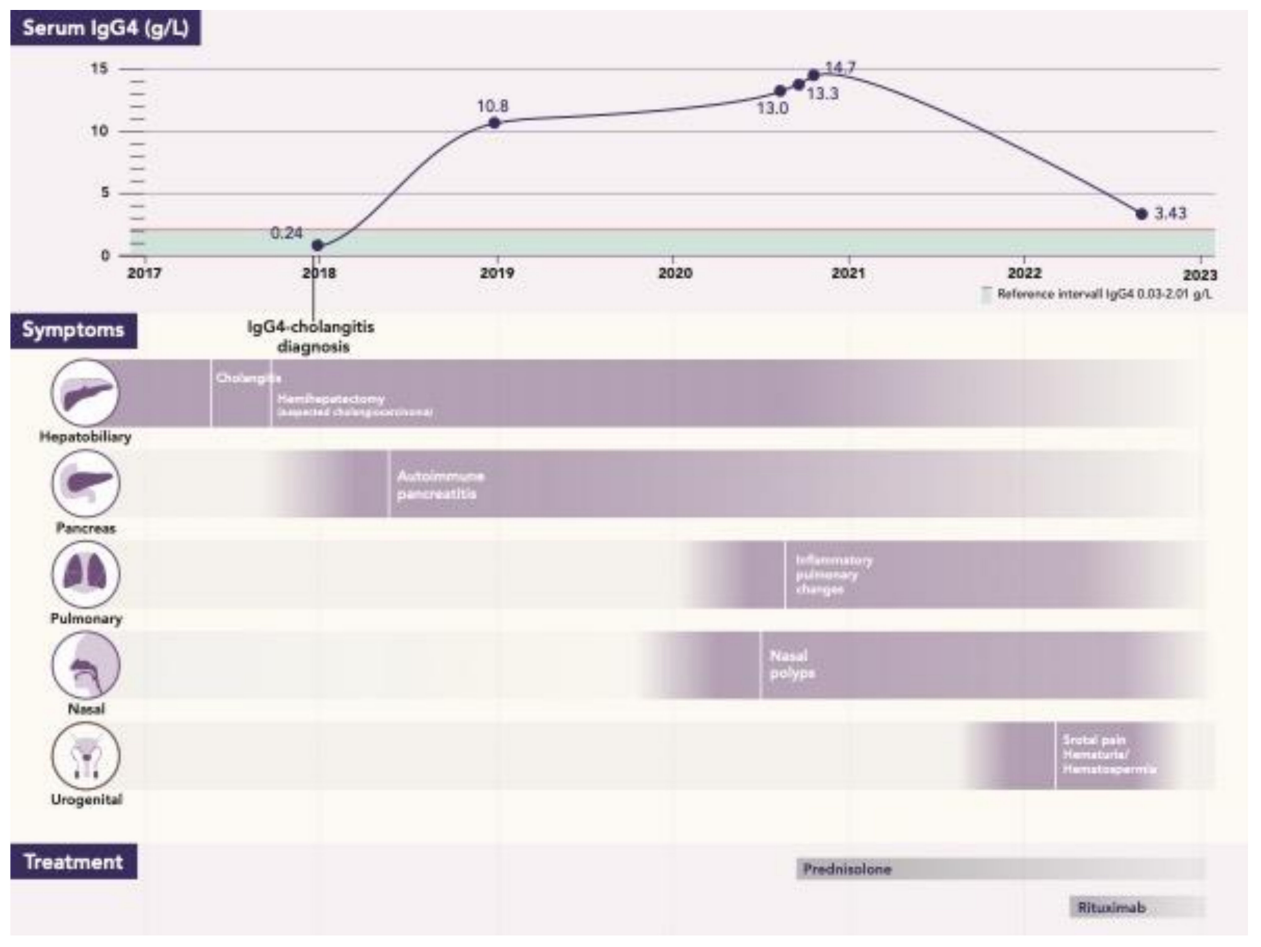
Case report: timeline with all events/parameters during the period of 2017–2022.

During 2020, the patient noticed a runny nose and a feeling of swelling in the nose and recalled that he had problems with nasal polyps 20 years prior. The patient was referred to otorhinolaryngologist, who found a small greyish polyp in the meatus media and sphenoidal compartment on the right side, as well as more polyps on the left side further back in the sphenoidal compartment, originating from ethmoidal cells. A biopsy was performed, and histopathology showed strongly elevated IgG4 plasma cells (>50 per high power field and IgG4/IgG ratio > 40). (Figure 1). Saline rinse and mometasonfuroat nasal spray were recommended. At the same time (2020), the patient experienced coughing with a little slime and was referred to a pulmonologist. A CT of the thorax was performed, which showed a spiculated infiltrate in the left lung apex that had increased in size considerably since 2017 (Figure 2). A CT-guided intermediate needle biopsy was performed, but the results were inconclusive. ^18^F-fluorodeoxyglucose (FDG) PET/CT showed uptake in the left apex, but it was not possible to differentiate between IgG4 inflammation and malignancy. There was no FDG uptake in the other organs. The serum IgG4 levels were at their highest (14.7 g/L) since 2017. Treatment with 40 mg prednisolone per day was started (before that, a control DXA was performed and showed unchanged osteopenia status; vitamin D and calcium were prescribed as prophylaxis during the steroid treatment). Control CT of the thorax showed significant improvement and regression of the previously described lesions. Prednisolone was tapered to 5 mg per week, and in January 2022, came to a maintained dose of 5 mg per day. In February 2022, the patient was referred to a urology outpatient clinic due to testicular pain, hematuria, dysuria, and fever up to 38 °C. The patient explained that hematuria was, in fact, one occasion of hematospermia. Furthermore, testicular tenderness/pain had been present periodically for at least 10–15 years without any progress, but he had never sought medical help for that. The patient estimated a normal miction frequency and dysuria consisting of a feeling of incomplete bladder emptying. At physical examination, in the standing position, scrotal inspection showed a higher standing right testicle with otherwise normal scrotal configuration. On palpation, both testicles revealed a hard consistency and somewhat uneven shape. The epididymites were tender and enlarged bilaterally, especially in the caudal parts. The distal parts of the funicles were slightly tender, and the patient had a right-sided inguinal hernia. The rectal examination and cystoscopy were normal, with normal bladder cytology. Ultrasound (US) showed aberrant heterogeneous parenchymal structure in the testicles bilaterally, more clearly on the left side, with an appearance of chronic post-inflammatory changes (Figure 3, Figure 4 and Figure 5). There were no signs of focal changes or malignancy. A CT of the kidneys and ureters showed no signs of malignancy in the urinary tract. The patient’s case was discussed with rheumatologists, and he started treatment with rituximab in May 2022. Urological control in September 2022 showed clinical improvement in all symptoms, as well as US (regression of inflammatory changes in testicles and epididymis) and laboratory improvement (serum IgG4 lowered to 3.4 g/L). Control at the pancreas outpatient clinic showed an asymptomatic patient with fecal elastase-1 levels in the normal reference interval (232 μg/g), but lower than the levels during the period of 2018–2020 (>500 μg/g). A graphical case report with the timeline and the most important findings is presented in Figure 6.

## 4. Literature Review

**Scheme 1:**
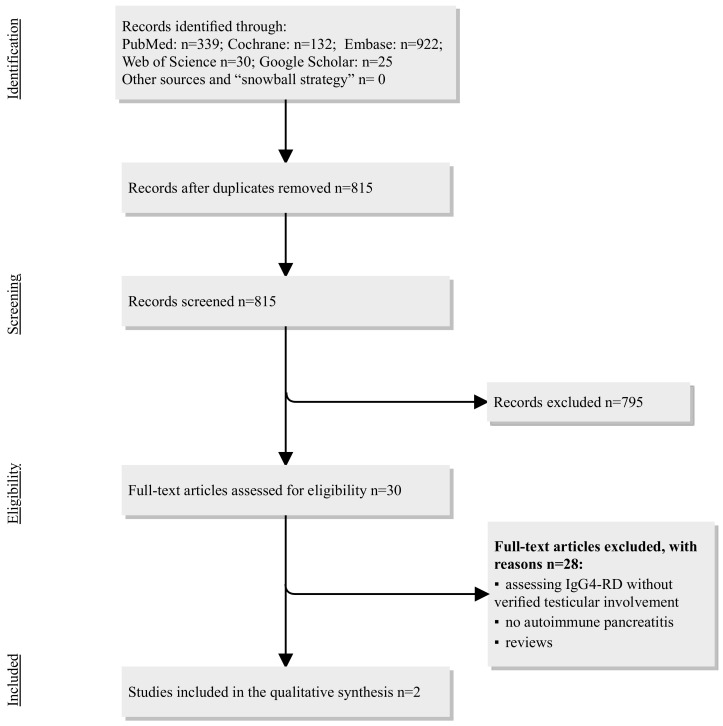
Flowchart of the study selection process regarding IgG4-RD in the pancreas and testicles. Abbreviations: IgG4-RD = immunoglobulin G subclass 4-related disease.

**Scheme 2:**
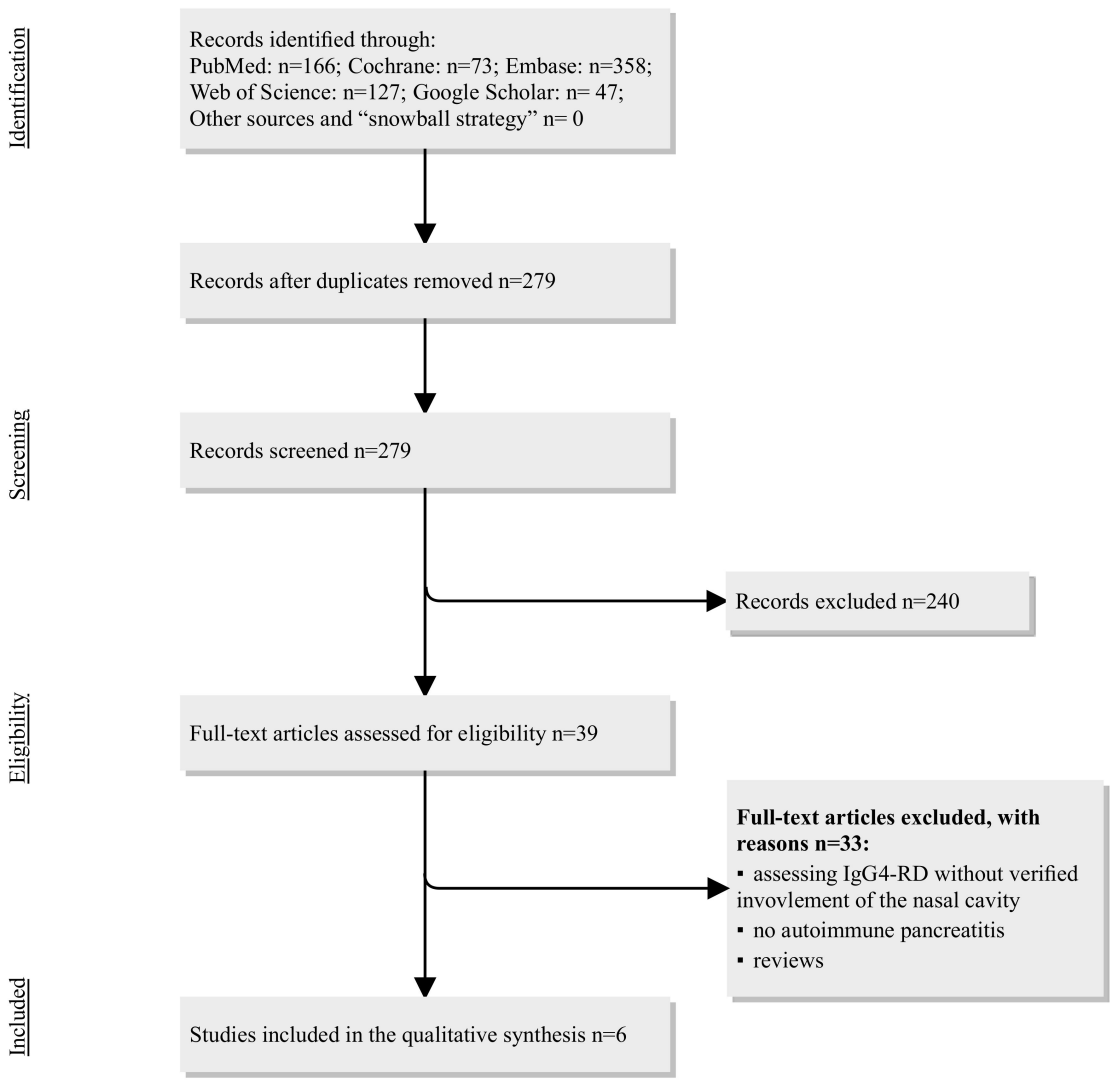
Flowchart on the study’s selection process regarding IgG4-RD in pancreas and sinonasal cavity. Abbreviations: IgG4-RD = immunoglobulin G subclass 4-related disease.

A comprehensive literature search for relevant articles was performed (Scheme 1 and Scheme 2) by four independent authors (M.K., D.B., N.P. and E.F.) in accordance with the Preferred Reporting Items for Systematic Reviews and Meta-Analyses (PRISMA) guidelines, and all disagreements were resolved by the senior authors (MV, JML). Embase, Google Scholar, PubMed, Cochrane, and Web of Science were searched using the following terms: (“Immunoglobulin G4-related disease” or “IgG4-related disease” or “Autoimmune pancreatitis type 1”) and (“testicular” or “testis” or “paratestis” or “orchitis” or “epididymis” or “scrotum”, or “scrotal” or “intrascrotal” or “spermatic cord” or “vas deferens” or “paratesticular pseudotumor” or “tunica vaginalis” or “genitourinary” or “urology”). The aforementioned databases were also searched using the following terms: (“Immunoglobulin G4-related disease” or “IgG4-related disease” or “Autoimmune pancreatitis type 1”) and (“nose” or “nasal cavity” or “nasal polyps” or “rhinitis” or “otorhinolaryngologic diseases” or “otolaryngology” or “otorhinolaryngology” or “paranasal sinuses” OR “nasal sinuses”). The search was limited to studies with human subjects and which had been published before 31 December 2022. Additionally, a manual examination of the references listed by the studies (“snowball method”) retrieved from the online databases and previously published reviews was performed in order to identify other potentially relevant studies for inclusion. We included articles assessing autoimmune pancreatitis type 1 (IgG4-RD) patients with testicular or nasal cavity involvement, with the full text available, published in the English language, with the diagnosis of IgG4-RD (definite or probable) in accordance with the 2020 revised comprehensive diagnostic criteria [11]. Literature pertaining only to IgG4-RD without testicular or nasal cavity involvement was excluded, as were cases of testicular and nasal IgG4-RD without AIP type 1 and those that reported pancreatic, testicular, and nasal cavity without specifying patients with overlapping involvement of all three organs. We focused only on (and limited our search to) cases of AIP type 1 with testicular and nasal involvement. Due to the relatively small number of studies on AIP type 1 with testicular involvement and nasal cavity involvement, which are rare in everyday clinical practice, even case reports were used. 

## 5. Results

### 5.1. AIP Type 1 and Testicular Involvement

The systematic review identified two case reports of AIP type 1 and testicular involvement (Table 1). In an article from the USA in 2012 [12], the authors report the case of a 67-year-old male with known IgG4-related disease (in the pancreas, retroperitoneal fibrosis, and bilateral kidney involvement) who presented five years later with a painless right scrotal mass gradually increasing in size. Owing to the potential risk of malignancy, albeit small, he underwent right inguinal radical orchiectomy with histopathologic and immunohistochemistry findings characteristic of IgG4-related disease. IgG4 in the serum was 391 mg/dL (reference < 140 mg/dL) at the time of initial IgG4-RD diagnosis, but the serum IgG4 at the time of testicular involvement was not reported. Another case was reported from the Netherlands, describing a 57-year-old patient with known AIP type 1 diagnosed after a Whipple operation due to suspected pancreatic cancer [13]. After seven years, this patient presented with scrotal pain and was treated with antibiotics for suspected epididymitis. An ultrasound showed inhomogeneous alterations, and the patient underwent a left orchiectomy due to suspected malignancy. A right orchiectomy was performed seven months later due to abscess-forming inflammation in the right testis. Histology in both specimens showed IgG4-RD.

### 5.2. AIP Type 1 and Nasal Cavity Involvement

The systematic review identified six studies, with a total of 143 patients included (Table 2). Two studies presented case reports [14,15], and four described case series [16,17,18,19]. Four studies were from Japan [14,16,17,19] one from China [18], and one from the USA [15]. The symptoms reported in sinonasal IgG4 engagement included nasal crusting, nasal blockage, secretions, and hyposmia. Moteki et al. evaluated steroid treatment in a cohort of 31 patients with IgG4-related disease and found 7 patients with both AIP and sinonasal engagement, presenting with nasal obstruction, rhinorrhea, postnasal drip, and anosmia, as well as elevated IgG4 in serum [16]. After approximately three months of prednisolone administration, AIP improved in six out of seven patients, serum IgG4 levels markedly decreased, and sinonasal symptoms improved. In a retrospective study, Suzuki et al. found 7 patients (out of 23 reviewed) with AIP and nasal involvement, who were treated with oral corticosteroids and intranasal corticosteroids. Unfortunately, no details of the outcome or follow-up were reported [19]. Cain et al. [15] and Ohno et al. [14] described case reports in patients with AIP and sinonasal engagement. Hanaoka et al. reviewed 108 patients with IgG4-related disease and found five patients with AIP and sinonasal engagement, in whom the chief nasal complaints were hyposmia and nasal obstruction [17]. The largest study so far was published recently by a group of Chinese authors, who performed a cross-sectional study on 408 patients with IgG4-related disease and comorbidity with allergic rhinitis, chronic rhinosinusitis, or both and found 122 patients (30%) who also had AIP [18]. Patients with allergic rhinitis and chronic rhinosinusitis in this study were sensitive to glucocorticoid therapy, and their nasal symptoms either were alleviated or disappeared after treatment, while chronic rhinosinusitis alone was insensitive to glucocorticoid therapy (information on treatment was available only for the whole cohort of patients in this study, and not for AIP separately). 

## 6. Discussion

We have described a unique case of a patient with IgG4-RD in the pancreas (autoimmune pancreatitis type 1) with involvement of the testicles, nasal cavity (nasal polyps), lungs, and bile ducts. Disease was diagnosed on the basis of classic HISORt (histology, imaging, serology, other organ involvement, and response to treatment) concept, first published in 2006 [20], and therefore confirmed under currently used pancreatological and radiological criteria [1,7,11]. Our group has already published data on kidney [10], biliary [21], lung [8], and cardiovascular [8] involvement, commonly occurring in patients with AIP type 1. However, this was the first patient in our historical cohort (one of the largest in Europe or the world) with both testicular and nasal involvement; therefore, we decided to perform a systematic review on the association between AIP type 1 and testicular and nasal involvement. As expected, the number of studies and cases was low. 

We found only two case reports on AIP and testicular involvement, and in both cases, orchiectomy was performed due to suspected malignancy (Table 1). In our patient, the diagnosis of IgG4-RD was histologically confirmed after abdominal surgery, which was performed due to suspected cholangiocarcinoma. Since AIP with immune-related cholangitis was known, as well as lung and nasal involvement, and especially after a scrotal ultrasound showed no suspected tumor, we suspected that the pathology in the testicles was also IgG4-RD. The diagnosis was indirectly confirmed with a complete clinical and radiological response after rituximab treatment. Teamwork is very important in cases of diagnostic uncertainty, because appropriate diagnosis and management can prevent unnecessary orchiectomy. Our case is also the only one amongst the few reported cases to be treated with rituximab. Epididymo-orchitis can occur in various forms of vasculitis (polyarteritis nodosa, Behçet’s disease, granulomatosis with polyangiitis, and sarcoidosis), but is often only treated with steroids. To the best of our knowledge, there are no reported cases of rituximab treatment for autoimmune epididymo-orchitis on a vasculitis base. The number of patients with nasal involvement was low until recently, when the largest study on this topic was published by a group of Chinese authors, who reported 122 patients with both pancreas and nasal involvement [18]. New onset of symptoms such as nasal blockage, rhinorrhea, sinusitis, hyposmia, crusting, or facial pain in a patient with IgG4-related disease should lead to suspected sinonasal comorbidity. It seems that sinonasal involvement in IgG4-related disease is more common without pancreatic involvement, and it has been proposed that sinonasal IgG4 could be considered a separate entity [16,17]. 

Interestingly, serum IgG4 in our patient was initially negative and only became elevated later in the course of the disease (Figure 6). A lack of sensitivity and specificity of the IgG4 serum level to establish the diagnosis of IgG4-related disease, or to distinguish it from other diseases, has previously been reported, and it seems that IgG4 serum levels have diagnostic value only when the level is higher than four times the upper level of normal, which is the case in only a minority of patients in everyday clinical practice. Thus, IgG4 serum levels alone are not accurate in the context of monitoring the disease course, nor do they sufficiently correlate with the development of complications or disease relapse [22,23,24]. The patient presented in this report is now in remission under the treatment with rituximab. Based on the results of our group, and those of the systematic review in [9], rituximab therapy seems to be effective in terms of inducing and maintaining remission of AIP type 1, with a low rate of side effects. According to the European Guidelines on IgG4-related digestive disease, glucocorticoids remain the most effective initial treatment (although there are limited clinical trials on the effectiveness of glucocorticoid maintenance therapy), and rituximab should be considered if patients are resistant or intolerant to high-dose glucocorticoids to maintain remission or have failed to respond to immunosuppressive therapies [25]. The rarity of reported testicular engagement together with other organ engagement in IgG4-RD, compared with other forms of vasculitis, could be due to the diagnostic challenge of performing a testicle biopsy in order to gain a histopathologically confirmed diagnosis. Other authors have also suggested that testicular symptoms might be present during an early stage of IgG4-RD and be misdiagnosed [26]. 

## 7. Conclusions

IgG4-RD can occur at almost any anatomical site, and often presents as a multiorgan disease that may mimic malignancy. In this paper, the first case of AIP type 1 with other organ involvement, including testicular and nasal involvement, has been described. The number of patients with nasal and testicular involvement described in the literature is low. Creating awareness about this rare clinical condition is necessary, especially due to the availability of a very effective treatment with corticosteroids and rituximab. 

## Figures and Tables

**Table 1:** Studies on patients with autoimmune pancreatitis type 1 (IgG4-related) and testicular involvement.

Author, Year, Country	Age	Medical History and Clinical Presentation	Treatment	Histology	Follow-Up
Hart et al. [12], 2012, USA	67	IgG4-RD in pancreas (histologically confirmed), retroperitoneal fibrosis causing left hydronephrosis and bilateral kidney involvement with renal failure. Patient was successfully treated with 3-month course of corticosteroids and ureteral stenting. At the same time, patient developed DM. Five years after IgG4-RD diagnosis, patient presented with painless right scrotal mass that had been gradually increasing in size for three months.	Due to the potential risk of malignancy, patient underwent right inguinal radical orchiectomy.	Grossly, para-testicular tissue was involved with cystic formation. Microscopy showed lymphoplasmacytic inflammatory infiltrate, storiform fibrosis, obliterative phlebitis, perineural inflammation, and IgG4 positive cells (>50/HPF and IgG4/IgG ratio of 60%). The testicular parenchyma was not involved.	A pancreas relapse was suspected, and corticosteroids were introduced postoperatively. However, magnetic resonance imaging showed no signs of active inflammation in the pancreas, so corticosteroids were tapered off.
de Buy Wenniger et al. [13], 2013, the Netherlands	64	IgG4-RD in pancreas (histologically confirmed after Whipple operation performed due to suspected pancreatic cancer). Seven years after IgG4-RD diagnosis, patient presented with scrotal pain.	Patient was initially treated with antibiotics for suspected epididymitis. Ultrasound showed inhomogeneous alterations, and the patient underwent left orchiectomy due to suspected malignancy. Right orchiectomy was performed seven months later due to abscess-forming inflammation in the right testis.	IgG4 staining of the tissue of the right testis showed a high number of infiltrating IgG4-positive cells around a seminiferous tubule (focally maximum 50/HPF). The earlier resected left testis showed similar changes (maximum 80 IgG4-positive cells/HPF).	Not reported.

IgG4-RD = immunoglobulin G4-related disease; HPF = high-power field.

**Table 2:** Studies on patients with autoimmune pancreatitis type 1 (IgG4-related) and nasal involvement.

Author, Year, Coutry	Age, Sex	Medical History and Clinical Presentation	Histology	Treatment	Follow-Up
Moteki et al. [16], 2011, Japan	Mean age 57.6 ± 6.5 years (range of 50–68) 6 males and 1 female	A total of 7 patients (out of 31 presented) had both autoimmune pancreatitis and nasal involvement. Patients had nasal problems including nasal obstruction, rhinorrhea, postnasal drip, and anosmia, and were diagnosed as having allergic rhinitis and/or chronic rhinosinusitis. All 7 patients had elevated IgG4 in serum: 2970, 387, 773, 1610, 1910, 209, 1500 (cut-off: 135 mg/dL)	Histology was performed in 5 patients. IgG4-positive plasma cell infiltration in nasal mucosa was present in all patients: severe in 4 (>30/HPF) and moderate in 1 (10–30/HPF).	In total, 70% of patients who had chronic rhinosinusitis were treated with oral prednisolone (40 mg per day for 4 weeks, tapered 5 mg per week). Other patients were not treated because of mild symptoms, unstable diabetes mellitus, or other comorbidities.	After approximately 3 months of prednisolone administration, at the point when it was possible to reduce the dosage to 5–15 mg/day, the characteristic findings of autoimmune pancreatitis had improved in six patients. Serum IgG and IgG4 levels were markedly decreased. Nose, nasal discharge, and obstruction symptoms were also improved.
Suzuki et al. [19], 2013, Japan	Mean age 67.3 ±8.9 years (range 55–79) 6 males and 1 female	A total of 7 patients (out of 23 presented) had both autoimmune pancreatitis and nasal involvement. Nasal symptoms included nasal obstruction, nasal crusting, and rhinorrhea.	High infiltration of IgG4 plasma cells (>50/HPF) in nasal mucosa in all 7 patients: 220.8 ± 106.5 (range of 101–391)	Oral corticosteroids and intranasal corticosteroids were used (no details reported)	NA
Cain et al. [15], 2014, USA	60, male	Nasal congestion, cervical lymphadenopathy, retro-orbital pain, and vision changes. MRI revealed a unilateral cavernous sinus-enhancing soft tissue mass. Serum IgG4 was elevated (195 mg/dL) Six months previously he had undergone a partial pancreatectomy for a pancreatic head mass, which was found to be autoimmune pancreatitis.	The pancreatectomy specimen showed focal lymphoplasmacytic infiltrate and marked sclerosis, consistent with IgG4-associated autoimmune pancreatitis.	Prednisone 40 mg daily, with rapid resolution of his symptoms. Transitioned from prednisone to azathioprine and continued to do well without relapse.	Repeat MRI after 1 month of prednisone showed a marked decrease in size of the previously noted cavernous sinus mass with minimal residual enhancement. This mass was therefore determined to be an inflammatory pseudotumor secondary to IgG4-RD. Surveillance MRI 2 years later showed complete resolution of the cavernous sinus mass.
Ohno et al. [14], 2015, Japan	55, male	Swelling of the bilateral submandibular glands (4 cm in diameter) and elevated serum IgG4 (514 mg/dL). One month later, hyposmia and diffuse, erosive, crusty lesions on nasal mucosa were found. Two years after admittance, the patient was diagnosed with autoimmune pancreatitis.	Biopsy from the submandibular gland and the inferior turbinate mucosa showed dense infiltration of IgG4-positive plasma cells and a high IgG4/IgG ratio (submandibular gland 220/HPF, 70%; nasal mucosa 100/HPF, 50%) with fibrosis.	Initially, patient was treated with oral prednisolone (start 0.6 mg/kg) with tapering (suspended after one year). Two years later, the patient was diagnosed with autoimmune pancreatitis and has been followed regularly without general steroid administration.	After prednisolone treatment, the nasal mucosa normalized and the hyposmia improved. Control biopsy of nasal mucosa improved (IgG4+ lowered to 3/HPF and IgG4/IgG ratio to 5%). Serum IgG4 persisted at a high level, independently of the dose of oral prednisolone.
Hanaoka et al. [17], 2017, Japan	Mean age 59.8 ± 9.3 years (range 48–69) 3 males and 2 females	A total of 5 patients (out of 108) had both autoimmune pancreatitis and nasal involvement. The chief nasal complaints were hyposmia and nasal obstruction.	Abundant infiltration of IgG4-positive plasma cells (>10/HPF) was found in 3 of 4 patients in whom histology was performed (45, 20, 190/HPF). In one patient, IgG4 was 8/HPF. Neither storiform fibrosis nor obstructive phlebitis was observed in any patient.	All patients were treated with steroid therapy. Endoscopic sinus surgery was performed in 4 of 5 patients.	No recurrence was noted.
Shi et al. [18], 2022, China	Mean age at disease onset was 56 years (range 46–62) Male:female = 1.34:1	122 patients (out of 408) had pancreas involvement. The main clinical nasal manifestations were sneezing, nasal obstruction, nasal itching, and rhinorrhea. Median serum IgG4 was 650 (312–1520) mg/dL.	A total of 46 patients underwent nasal tissue biopsies, and among them, 28 patients had dense IgG4-positive plasma cells (>10/HPF) and a ratio of IgG4/IgG of > 40%. The other 4 patients had storiform fibrosis and/or obliterative phlebitis.	Patients with allergic rhinitis and chronic rhinosinusitis were sensitive to glucocorticoid therapy, and their nasal symptoms alleviated or disappeared after treatment, while chronic rhinosinusitis alone is insensitive to glucocorticoid therapy (information on treatment is available only for the whole cohort of patients in this study).	Follow up period was 51.5 (33–72) months.

MRI = magnetic resonance imaging; IgG4-RD = immunoglobulin G subclass 4-related disease; NA = not available; HPF = high-power field.

## Data Availability

The data collected and analyzed during the current study are available from the corresponding author upon reasonable request.

## Abbreviations

AIP = autoimmune pancreatitis; CT = computed tomography; ERCP = endoscopic retrograde cholangiopancreatography; FDG = fluorodeoxyglucose; IgG4 = immunoglobulin G subclass 4; IgG4-RD = IgG4-related disease; MRI = magnetic resonance imaging; PET = positron emission tomography; PRISMA = Preferred Reporting Items for Systematic Reviews and Meta-Analyses; US = ultrasound.
